# Priority without progress: the FDA's neglected tropical disease voucher program after 18 years

**DOI:** 10.1093/haschl/qxag024

**Published:** 2026-01-29

**Authors:** Maple Goh, Kevin Outterson, Aaron S Kesselheim

**Affiliations:** Program On Regulation, Therapeutics, And Law (PORTAL), Division of Pharmacoepidemiology and Pharmacoeconomics, Department of Medicine, Brigham and Women's Hospital/Harvard Medical School, Boston, MA 02120, United States; CARB-X, Boston University School of Law, Boston, MA 02215, United States; Department of Community Medicine and Public Health, Tufts University School of Medicine, Boston, MA 02111, United States; CARB-X, Boston University School of Law, Boston, MA 02215, United States; Program On Regulation, Therapeutics, And Law (PORTAL), Division of Pharmacoepidemiology and Pharmacoeconomics, Department of Medicine, Brigham and Women's Hospital/Harvard Medical School, Boston, MA 02120, United States

**Keywords:** neglected tropical diseases, drug approval, World Health Organization, priority review vouchers, health policy, global health

## Abstract

**Introduction:**

To incentivize drug and vaccine development for neglected tropical diseases (NTDs), US Congress created the Priority Review Voucher (PRV) program in 2007. Sponsors that obtain Food and Drug Administration (FDA) approval for an eligible product receive a voucher redeemable to accelerate review of another product.

**Methods:**

We reviewed the program's public health impact by examining all 14 vouchers awarded for NTD products between 2007 and 2024, including the timing of FDA approval relative to World Health Organization (WHO) Prequalification, Essential Medicines List inclusion, first use in endemic countries, and voucher disposition.

**Results:**

Eight (57%) achieved WHO Prequalification, and 8 (57%) were listed in the Essential Medicines list. FDA approval occurred a median of 8.7 years after first regulatory approval or use in an endemic country and a median of 5.2 years after WHO Essential Medicines list inclusion.

**Conclusion:**

Our findings suggest that the PRV program has primarily rewarded regulatory filings for long-established therapies rather than stimulating innovation or improving access. We propose reforms linking voucher eligibility to equitable pricing and endemic country registration.

Key points

- FDA approval occurred a median of 8.7 years after first regulatory approval or use in an endemic country.
- The FDA's neglected tropical disease voucher program has primarily rewarded regulatory filings for long-established therapies with limited alignment on global access milestones.
- Program reforms should link voucher eligibility to equitable pricing, registration in endemic countries, and demonstrable access gains to ensure incentives align with public health impact.

## Introduction

Neglected tropical diseases are a group of infectious diseases that disproportionately affect populations in low- and middle-income countries and therefore historically receive limited investment in research and development from private manufacturers.^1^ While the highest neglected tropical disease burden remains in western sub-Saharan Africa, the last 3 decades have seen middle, high-middle, and high social development index regions experience an increase in age-standardized incidence rates of neglected tropical diseases.^2^ Brazil, India, Indonesia, Mexico, and even the United States^3^ now harbor a significant share of the global neglected tropical disease burden.^4^ It is estimated that neglected tropical diseases affect more than 1 billion people globally^1^ and at least 12 million in the United States alone.^5^ Age-standardized, disability-adjusted life-years for total neglected tropical diseases in 2019 was estimated to be 13.18 million.^6^

To incentivize industry engagement in neglected tropical disease drug and vaccine development, US Congress established the Priority Review Voucher program in 2007.^7^ Sponsors can qualify for a priority review voucher by achieving US Food and Drug Administration (FDA) approval of a product that treats or prevents a tropical disease.^8^ The voucher entitles the bearer to force the FDA to review another product via the priority review pathway with a 6-month deadline when that product would otherwise have been subject to standard review with a 10-month deadline.^7^ Vouchers may be retained for internal use, sold, or transferred an unlimited number of times to other companies before they are redeemed.^7,9^ The value of the 4-month shorter deadline was estimated to be worth upwards of $300 million for manufacturers as an incentive to invest in tropical disease product development, although vouchers have more recently sold for approximately $100 million.^10^

As of 2016, 28 diseases can support a tropical disease voucher.^8^ Many of these overlap with the World Health Organization’s (WHO's) definition of neglected tropical diseases,^1,9^ such as tuberculosis, malaria, cholera, and Zika virus.^8^ While the voucher program was designed to stimulate industry engagement in neglected tropical disease product development, its public health impact depends on whether these products reach affected populations in a timely manner. Prior studies have found that several early priority review voucher awards followed US regulatory approval of therapies already in longstanding use in endemic countries.^11,12^ Inclusion in WHO policy instruments, such as the WHO Prequalification program and Essential Medicines List, can also facilitate global availability.^13^ The WHO Prequalification program serves as a gatekeeper for quality assurance and its inclusion is a minimum requirement for most major global health procurement agencies. An external impact assessment estimated that prequalification enabled $3.5 billion in donor-funded procurement annually, reaching approximately 400 million additional patients each year.^13-15^ Similarly, inclusion in the WHO Essential Medicines List, a register of minimum medicine requirements for every health care system and first established in 1977, has a direct effect on national drug formularies, financing, and procurement.^16^ Countries use the Essential Medicines List as a template for their own lists, and donor agencies require listing to approve grants and tenders. Studies have shown that medicines added to the list saw bulk purchasing, expanded insurance coverage, and formal inclusion in procurement systems.^17^ Conversely, its absence slows or prevents adoption and purchase, increases out-of-pocket expenditures, and reduces coverage.^17^

However, the extent to which voucher-awarded products achieve early adoption in endemic settings remains unclear. Prior analyses have largely focused on the economic value of vouchers and their potential to incentivize research and development,^10,12,18,19^ with limited attention to postapproval regulatory trajectories or uptake.^20^ Similarly, little is known about the timing of FDA approval relative to first approval in endemic countries, or whether certain product types are more likely to receive FDA approval first. To address these gaps, we conducted a comprehensive review of all neglected tropical disease priority review vouchers awarded from the program's inception in 2007 through 2024. We examined the following: (1) the timing from FDA approval to WHO Prequalification and Essential Medicines Listing; (2) the sequence of FDA approval and first approval in an endemic country; and (3) the disposition, resale patterns, and sale values of vouchers.

## Data and methods

This study evaluated markers of global access for drugs and vaccines awarded a neglected tropical disease priority review voucher by the FDA from 2007 through 2024. As the primary measures of public health impact, we examined the timing of each product's listing in the WHO Prequalification database and the WHO Essential Medicines List and first approval or use in an endemic country. We also evaluated the disposition and resale patterns of the vouchers awarded under the program.

Regulatory approval dates in the United States and endemic countries were identified using multiple sources, including PharmaProjects, the Drugs@FDA database, public Securities and Exchange Commission filings, press releases, annual reports, and other public statements from the manufacturer, and targeted literature searches. When discrepancies were identified between sources, the earlier date was used if other evidence confirmed that the product was already in use in that country. When only launch dates were available, these were used as a proxy for approval dates, acknowledging that launch dates occur after regulatory approval.

The definition of an endemic country was tailored to each disease, based on literature-derived definitions of endemicity. To qualify as the “first endemic country” in our analysis, the country had to be endemic for the condition at the time of approval or documented use. Endemicity status was verified through literature searches in PubMed (using the terms “[disease name]” AND “[country]” AND “endemic”) and consultation of WHO databases.

The US approval dates and priority review voucher issuance were found using the FDA Drugs@FDA database. The WHO Prequalification database was used to identify whether and when each product achieved prequalification status, and the WHO Essential Medicines List archives were reviewed to determine initial inclusion dates, subsequent revisions, and whether the product remained listed in the most recent edition. For vaccines, if the WHO Essential Medicines List recommended vaccination for the relevant disease at all, the product would be included in the time interval using calculations from the year that vaccine class was first included, regardless of whether the specific FDA-approved product was named. However, unless the product specifically was named, it was not considered as included in the list for the purposes of binary classification. This approach allowed us to capture the broader public health recognition of vaccine importance while distinguishing products that had achieved formal WHO endorsement.

PharmaProjects, public statements, annual reports, and FDA approvals were reviewed to identify the original developer of the product, priority review voucher sale value, and evidence of reinvestment into product access or rollout. The priority review voucher holder was classified as either a small- or medium-sized enterprise or a large enterprise according to the US Small Business Administration and matched to North American Industry Classification System codes.^21^ Using code 541715, small- or medium-sized businesses were defined as employing fewer than 1000 full-time equivalent employees. Employee counts were determined from publicly available sources, including company reports, press releases, and regulatory filings. In cases when a smaller firm was partnered with, acquired by, or otherwise directly affiliated with a large enterprise at the time of FDA approval, the sponsor was classified as a large enterprise for the purposes of this analysis. This approach was used to reflect the combined resources, market leverage, and strategic decision-making capacity available to the innovator at the time of earning the priority review voucher.

The primary outcomes were the number of years between FDA approval and WHO Prequalification and Essential Medicines List listing. Negative intervals indicated that the listing occurred before FDA approval. Secondary outcomes included the entity that redeemed the priority review voucher and the voucher sale value.

Descriptive statistical methods were used to summarize timelines and WHO Prequalification and Essential Medicines List inclusion. Time-to-event measures were calculated in days and expressed in years rounded to 1 decimal place.

## Results

### WHO Prequalification and Essential Medicines List listing timelines

Fourteen drugs and vaccines were approved by the FDA for indications qualifying under the neglected tropical disease Priority Review Voucher program between 2007 and 2024. Of these, 8 (57%) products achieved WHO Prequalification and 8 (57%) were included in the Essential Medicines List. Drugs with longstanding use and FDA approval, such as benznidazole, triclabendazole, and nifurtimox, are still not on the WHO Prequalification list. All of these drugs, however, are on the Essential Medicines List. Of the products with WHO Prequalification, the majority (5 [63%]) were listed after FDA approval, whereas for Essential Medicines List inclusion, nearly all (8 [89%]) achieved that status before FDA approval. Artemether-lumefantrine, for example, was on the Essential Medicines List in 2002, was added to the Prequalification list in 2004, and was FDA-approved in 2009. None of the vaccines in our cohort (Vaxchora, Dengvaxia, Ervebo, Ixchiq) were listed on the Essential Medicines List, because while the list recommends vaccination for cholera and dengue, it did not list the FDA-approved versions as the recommended product; for example, the Essential Medicines List refers to Qdenga as the recommended dengue vaccine, and not Dengvaxia.

**Figure 1.**
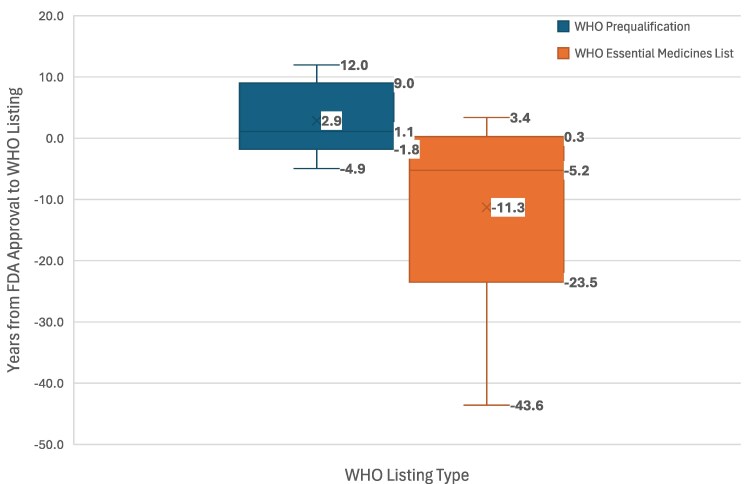
Time from FDA approval to WHO Prequalification or Essential Medicines List. Source: Authors' analysis of data from Drugs@FDA, WHO Prequalification, and Essential Medicines List. Abbreviations: FDA, Food and Drug Administration; WHO, World Health Organization.

The median time from FDA approval to WHO Prequalification was 1.1 years, but to Essential Medicines List was −5.2 years, indicating that, for most products, Essential Medicines List status preceded FDA approval (Figure 1). When miltefosine was first discovered by 2 independent research groups in the early 1980s, it was initially developed as an anticancer agent.^22^ It received its first approval in 1993 for the treatment of skin metastases in patients with breast cancer in Germany, followed by various other European countries.^23^ Clinical trials starting in 1996 led to its first regulatory approval granted in India in 2002.^22,23^ Fewer than 10 years later, it was listed on the WHO Essential Medicines List, and in 2014 was approved by the FDA. Similarly, benznidazole was first described as having trypanocidal activity in 1967.^24^ It was then introduced by Roche for Chagas disease, caused by *Trypanosoma cruzi*, in 1971.^25^ It first appeared in the Essential Medicines List in 1987, 30 years prior to its FDA approval in 2017.

### Regulatory approval timelines

**Figure 2.**
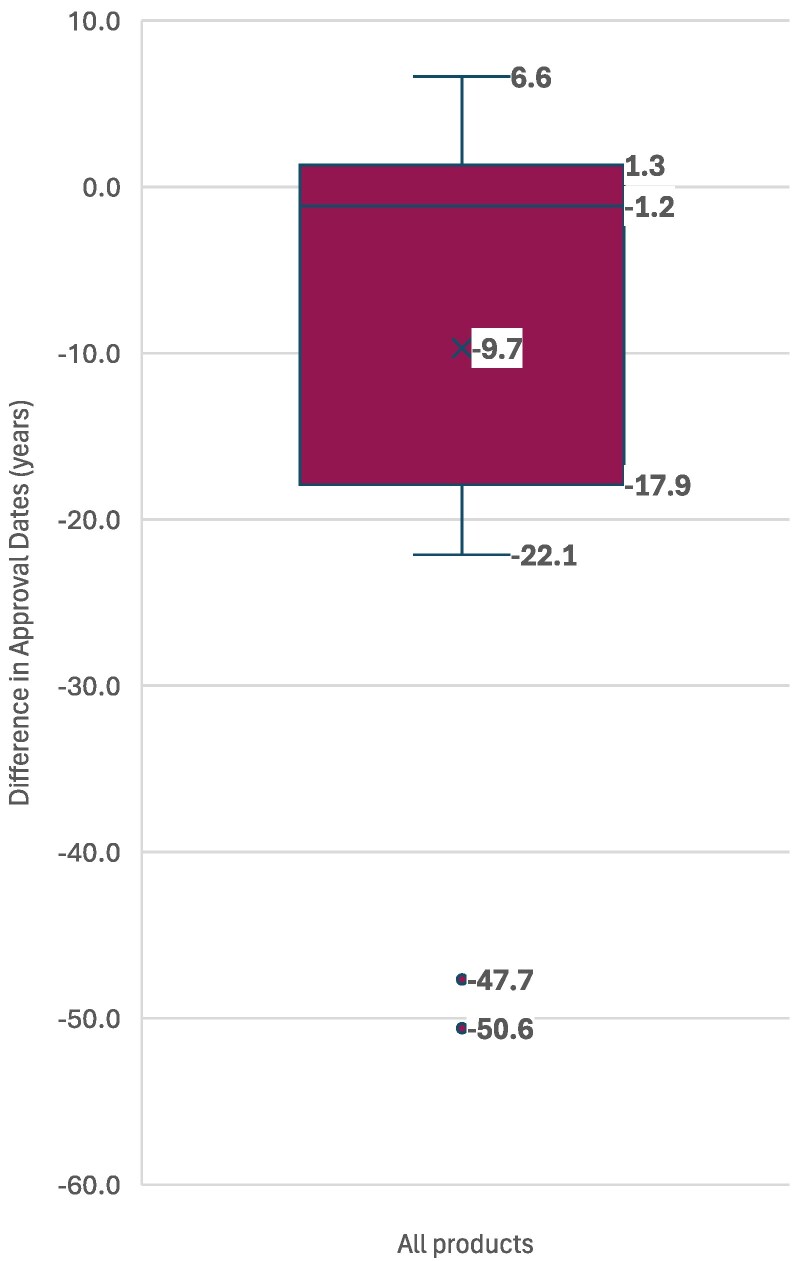
Years between FDA approval and first endemic country approval or use. Source: Authors' analysis of data from Drugs@FDA and literature review on endemic country approval or use. Abbreviation: FDA, Food and Drug Administration.

The median difference between FDA approval and first regulatory approval in an endemic country was −9.7 years, indicating that, in many cases, endemic countries had access to the product substantially earlier than the US market (Figure 2). Most strikingly, benznidazole and nifurtimox (−47.7 and −50.6 years) were used in endemic countries decades before their US approval. After benznidazole's launch in 1971, Roche maintained benznidazole production across multiple countries, including Brazil, Argentina, Bolivia, Uruguay, Peru, Nicaragua, and Japan.^26^ It received FDA approval in 2017, almost half a century later. Similarly, nifurtimox, first marketed in the 1970s, achieved FDA approval for pediatric use in 2020. Triclabendazole, first approved by the FDA in 2019, has also been known to be used since 1986,^27^ more than 3 decades prior to US approval.

Thus, approximately half of the products had been approved or used in at least 1 endemic country before US approval. Among the 7 products that received first global regulatory approval in the United States with no clear prior record of approval or use in an endemic country, 3 were among the 4 vaccines in our cohort (Vaxchora, Ervebo, and Ixchiq). Yet, these vaccines were not necessarily novel. For example, Vaxchora, while the first cholera vaccine licensed in the United States, is a reformulated version of CVD 103-HgR, which had previously been licensed as Orochol in Europe in the 1990s before being withdrawn due to low demand.^28^ Other oral cholera vaccines (Dukoral, Shanchol) were already in global use prior to Vaxchora's approval. The remaining 4 (bedaquiline, moxidectin, tafenoquine, pretomanid) were therapeutics. For example, moxidectin, which was first approved for human use in river blindness in 2018, had been widely used in veterinary medicine as an antiparasitic since the 1990s.^29^ Its subsequent development for human use involved new formulations, dosing, and administration routes, but it is the same active compound that has been used in veterinary medicine for decades for the same general category of disease (antiparasitic). Only bedaquiline and pretomanid are currently listed on the WHO Essential Medicines List.

### Voucher disposition, resale patterns, and impact

Of the 14 neglected tropical disease priority review vouchers awarded during the study period, 9 (64%) were awarded to large enterprises. Four of these 9 vouchers were the result of joint ventures between small- and medium-sized enterprises and large enterprises involving nonprofit–industry partnerships (Table 1). Almost all voucher sales with known buyers were to large enterprises. Reported sales prices ranged from $60 million to $290 million.

**Table 1.** Neglected tropical disease vouchers: originators, sponsor size, buyers, and values.

Product	Originator	SME or LE	Voucher buyer	Sale value (US$)
Artemether/lumefantrine (Coartem)	Novartis	LE	Used internally	n/a
Bedaquiline (Sirturo)	Janssen/Johnson & Johnson	LE	Used internally	n/a
Miltefosine (Impavido)	Paladin Labs (later Knight Therapeutics)	SME	Gilead Sciences	125 million^30^
Cholera vaccine, live, oral (Vaxchora)	PaxVax	SME	Gilead Sciences	290 million^10^
Benznidazole	Drugs for Neglected Diseases Initiative, Insud Pharma's Chemo Research, Mundo Sano Foundation	LE	Novo Nordisk	Not known
Moxidectin	Medicines Development for Global Health	SME	Novo Nordisk	Not known
Tafenoquine (Krintafel)	GlaxoSmithKline and Medicines for Malaria Venture	LE	Used within company at ViiV Healthcare	n/a
Triclabendazole (Egaten)	Novartis	LE	Used internally	n/a
Dengue tetravalent vaccine, live (Dengvaxia)	Sanofi	LE	Used internally	n/a
Pretomanid	TB Alliance	SME	Not known	105 million^10^
Ebola vaccine, live (Ervebo)	Merck and NewLink Genetics (Lumos Pharma)	LE	Used internally at Merck (Lumos paid $60 million)	n/a^10^
Nifurtimox (Lampit)	Bayer	LE	Not known	n/a
Fexinidazole	Drugs for Neglected Diseases Initiative and Sanofi	LE	Used within company at Sanofi	n/a
Live-attenuated chikungunya vaccine (Ixchiq)	Valneva	SME	Novartis	103 million^31^

Source: Authors' analysis of data from Drugs@FDA, public sources, press releases, and literature.

Abbreviations: LE, large enterprise; n/a, not applicable; SME, small- and medium-sized enterprise.

For example, the WHO Special Program for Research and Training in Tropical Diseases partnered with Asta Medica (later Zentaris) to develop miltefosine for visceral leishmaniasis in 1995.^22^ The drug's ownership was sold to Paladin Labs for $8.5 million in 2008.^22^ Knight Therapeutics received ownership from Paladin in 2014, and has since owned worldwide rights related to its sale and distribution in all countries other than the United States.^22^ Knight Therapeutics collected information from existing studies^32^ and submitted it to the FDA to receive approval on March 19, 2014, and a neglected tropical disease priority review voucher—12 years after the drug's first global regulatory approval—which it sold to Gilead for $125 million.^30^

Across multiple drugs, high drug costs have persisted even after FDA approval and voucher issuance. In the United States, a full drug course of miltefosine at the average wholesale price of miltefosine for an average 70-kg individual was US$48 000.^3^ In Germany, 1 course costs €3000–€12 000 (US$3500–$14 000).^33^ By contrast, in Pakistan, an equivalent full course costs PKR375 (US$1.33).^34^ In recent years, the drug has experienced frequent shortages. In Asia, national programs faced bureaucratic procurement hurdles, minimum order requirements of up to 200 000 capsules, and long manufacturing lead times, all of which contributed to frequent shortages and drug wastage.^22^ Registration has reportedly lapsed in countries across Africa and Latin America due to the reluctance of the manufacturer to register the drug in endemic countries.^22,35^

Similarly, while approximately 300 000 persons in the United States are infected with *T cruzi*, the protozoan causing Chagas disease,^36^ fewer than 1% were estimated to receive treatment with benznidazole or nifurtimox between 2007 and 2013.^37^ Post–approval and voucher issuance, the mean number of persons who obtained benznidazole only increased from under 5 to 13 per month.^38^ A 2020 study identified 5 key barriers shaping access in the United States: (1) no national network existed for multisector collaboration and fragmentation of existing groups; (2) order form requirements and a lack of an emergency delivery system with frequent delays in dispensing prescriptions; (3) high out-of-pocket costs for uninsured patients; (4) narrow indications for children, limiting promotion and demand among adult patients, and a lack of clinical practice guidelines for Chagas; and (5) the prescription of benznidazole for patients without a confirmed diagnosis or ineligible patients.^38^ In endemic Latin America, chronic structural supply issues continue to be documented.^39,40^

## Discussion

The neglected tropical disease Priority Review Voucher program was established with the intent of stimulating investment in drug and vaccine development for infectious diseases prevalent in low-income settings around the world that have been historically overlooked by the for-profit pharmaceutical sector. Our analysis of products awarded vouchers between 2007 and 2024 reveals that, although the program has generated some substantial financial transfers to product sponsors, these sponsors were not usually involved in the discovery of the drug and the voucher program's overall impact on global health appears minimal.^26^

Although most products associated with the Priority Review Voucher program were widely used around the world prior to FDA approval, some also achieved substantial public health impact after FDA approval. For example, bedaquiline, which emerged from a partnership between the pharmaceutical manufacturer Tibotec (an affiliate of Janssen) and the TB Alliance (with funding from the Gates Foundation, USAID, and others), was approved by the FDA in 2012 and the European Medicines Agency in 2013. It has been widely adopted for the treatment of multidrug-resistant tuberculosis, with approximately 800 000 treatment courses delivered across more than 150 countries since that time.^41^

However, a central finding is the substantial length of time between first global approvals of many neglected tropical disease therapies and their eventual FDA approval, which then triggered voucher eligibility. Miltefosine, benznidazole, nifurtimox, triclabendazole, and artemether-lumefantrine had all been in clinical use in endemic regions for more than a decade before FDA approval, yet sponsors were still awarded a voucher.^11^ Rather than incentivizing novel therapeutic breakthroughs, in these cases, the program has rewarded US regulatory filing prowess for long-established therapies.^8,11^

Voucher disposition patterns further underscore this misalignment. For miltefosine, the $125 million windfall to Knight Therapeutics had no discernible effect on resolving chronic shortages, registration gaps, or price disparities across South Asia, Latin America, and Africa.^22,30^ Similarly, US commercialization of benznidazole after FDA approval failed to meaningfully expand access in the United States. Across the cases, persistent challenges emerged: single-source manufacturing and fragile supply chains, lack of emergency delivery systems, complex order and prescribing requirements, and weak clinical guidelines in both endemic and nonendemic settings.^25,37,38^ These barriers reflect structural deficiencies in health systems and procurement mechanisms not resolved by the one-time financial incentives of the voucher program.

Our timeline analysis also showed that inclusion in the WHO Essential Medicines List and Prequalification often preceded FDA approval by several years, and many therapies were already approved or used in endemic countries before US licensure. This finding challenges the assumption that FDA approval acts as a gateway to global access. In fact, for several products, endemic populations had access decades earlier, while FDA approval primarily served to unlock voucher eligibility rather than to address unmet needs. The fact that 57% of voucher-associated products achieved WHO Essential Medicines List inclusion suggests that the voucher is ultimately attached to drugs of public health relevance. However, the timing, sequence, and conditions under which these milestones were reached are important considerations in interpreting the usefulness of the program.

Given concerns about the implementation of program raised by the FDA,^42^ the record from the last 15 years suggests that the neglected tropical disease Priority Review Voucher program should be repealed. If policymakers instead wanted to reform the program, they could consider measures to help realign financial incentives with public health outcomes. For example, Congress could condition voucher awards or redemption on demonstrable commitments to equitable access, such as registration in endemic countries, affordable pricing, or contributions to WHO prequalification; excluding products with long histories of clinical use outside the United States from eligibility; and enhancing transparency around the use of voucher proceeds.

This study was limited in that it relied on publicly available regulatory filings, WHO databases, and secondary literature, which may not capture confidential pricing, licensing, or access agreements. Sale values for several vouchers remain undisclosed. Our analysis focused on regulatory outcomes and did not examine downstream epidemiological impacts of product uptake, which warrant further study.

Our findings add to a growing body of literature questioning whether the neglected tropical disease Priority Review Voucher program achieves any public health goals.^9,11,18^ While it has provided substantial revenue to a small number of product developers, there is little evidence that the prospect of these funds led to innovation in the treatment options for neglected tropical diseases or even access gains for currently available therapies.

## Supplementary Material

qxag024_Supplementary_Data

## Data Availability

Data can be made available upon direct request from the authors.
